# Lactate and lactylation in liver diseases: energy metabolism, inflammatory immunity and tumor microenvironment

**DOI:** 10.3389/fimmu.2025.1581582

**Published:** 2025-05-12

**Authors:** Shiyi Cheng, Xiaoyu Xiao, Dingye Wang, Xiyuan Wang, Minlan Yang

**Affiliations:** School of Medicine, Yangtze University, Jingzhou, China

**Keywords:** lactate, lactylation, liver diseases, metabolism, immunology

## Abstract

Liver diseases pose a significant threat to human health. Lactate, a byproduct of glycolysis, serves various biological functions, including acting as an energy source, a signaling molecule, and a substrate for lactylation. Lactylation is a novel lactate-dependent post-translational modification that plays a role in tumor proliferation, the regulation of immune cell function, and the modulation of gene expression. In this paper, we summarize the roles of lactate and lactylation in energy metabolism, inflammatory immunity, and the tumor microenvironment, while also elucidating recent research advancements regarding lactate and lactylation in the context of hepatic fibrosis, non-alcoholic fatty liver disease, and hepatocellular carcinoma. Furthermore, lactate and lactylation are proposed as promising new targets for the treatment of liver diseases.

## 1 Introduction

Lactate (C₃H₆O₃) is the end product of glycolysis in the cytoplasm, where glucose is converted to pyruvate. This process is followed by the reduction of pyruvate to lactate, catalyzed by lactate dehydrogenase (LDH) (1). Under normal circumstances, lactate is continuously produced through glucose metabolism in the human body. However, its levels are regulated by metabolic processes such as complete oxidation, the Cori cycle, and participation in fatty acid synthesis, all of which help maintain acid-base homeostasis in the internal environment (2). Excessive accumulation of lactate in the bloodstream can lead to lactic acidosis, resulting in symptoms such as accelerated respiration, abdominal pain, nausea, dizziness, and drowsiness. The hypothesis proposed by Professor George Brooks in 1985 posits that during exercise, muscle cells rapidly produce and accumulate lactate (3). Some of this lactate is transported through the bloodstream to the heart, liver, and kidneys, where it is utilized through gluconeogenesis and other metabolic processes (4). The lactate shuttle is categorized into intercellular and intracellular lactate shuttles, primarily mediated by the monocarboxylate transporters (MCTs), which are expressed in organs such as skeletal muscle, the heart, and the liver (5). In cases of liver disease, impaired liver function leads to a decrease in lactate clearance, resulting in excessive lactate accumulation. This accumulation can exacerbate liver diseases, creating a vicious cycle. In 2019, a study from the University of Chicago first proposed that lactate acts as a post-translational modification of histones, influencing gene transcriptional regulation (6). Histone lysine lactylation (Kla) is a specific type of post-translational modification in which lactoyl groups are covalently attached to lysine residues of proteins, thereby facilitating gene regulation. This modification is also referred to as lysine lactylation or lactylation (6). Accumulated lactate is converted into Lactyl-Coenzyme A (Lactyl-CoA) as a direct substrate, catalyzed by a specific enzyme that facilitates the addition of lactate groups to designated modification sites on proteins, thereby completing the process of lactylation (2). Lactylation is characterized by its dependence on enzymes, a diverse array of modification sites, and competition with acetylation modifications (7). Additionally, lactylation plays multiple biological roles in regulating metabolic reprogramming, tumor progression, and immunomodulation. This review elucidates the biological functions of lactate and lactylation, and systematically summarizes their effects on liver diseases, including liver fibrosis, non-alcoholic fatty liver disease (NAFLD), and hepatocellular carcinoma (HCC). Furthermore, the potential therapeutic targets and clinical applications of lactate and lactylation as treatment options for liver diseases are discussed.

## 2 Biological functions of lactate

### 2.1 Lactate regulates lipid and glucose metabolism

Lactate was once regarded as a metabolic waste product under hypoxic conditions; however, subsequent research has demonstrated that lactate not only functions as an energy substrate but also plays a crucial role in regulating cellular metabolism (8). It primarily influences the activity and expression of enzymes involved in fatty acid and glucose metabolism, thereby impacting various metabolic processes and gene expression.

Acetyl-CoA carboxylase (ACC) is a crucial enzyme in fatty acid synthesis, and lactate enhances fatty acid metabolism by increasing ACC activity (4). G-protein-coupled receptor (GPR81) is a type of lactate receptor that primarily functions in adipocytes. Lactate inhibits lipolysis through the activation of GPR81 and the inhibition of the cyclic adenosine monophosphate-protein kinase A (cAMP-PKA) pathway, thereby reducing the release of free fatty acids (9). Additionally, lactate has been shown to increase the expression of the lactate transporter protein SLC5A12 in CD4^+^ T cells, facilitating lactate entry into the cell, which in turn promotes fatty acid synthesis and decreases glycolysis (10). Lactate regulates glucose metabolism in accordance with cellular energy demands and oxygen availability, thereby maintaining a stable intracellular environment. It primarily exerts its effects by modulating the activity of glycolysis-related enzymes and reducing the glycolytic rate, thus creating a feedback inhibition loop. 6-phosphofructo-1-kinase (PFK) is a key enzyme in glycolysis, and lactate binds to the allosteric site of PFK, diminishing its affinity for the substrates ATP and fructose-6-phosphate. This interaction decreases PFK activity and slows the rate of glycolysis (11). Furthermore, lactate inhibits the activities of hexokinase and pyruvate kinase, leading to reduced glucose consumption (12).

Lactate plays a crucial role in regulating lipid and glucose metabolism. In regulating lipids, it primarily promotes fatty acid synthesis while inhibiting lipolysis. Regarding glucose metabolism, lactate provides feedback that decreases the rate of glycolysis, thereby preventing excessive accumulation of lactate. Research on the regulation of lactate’s metabolic processes is limited, and future studies aim to uncover the detailed molecular mechanisms involved.

### 2.2 Lactate regulates inflammation and immunity

Lactate serves not only as a signaling molecule that participates in the regulation of inflammatory immune responses, but it also influences the function of immune cells and the expression of inflammatory factors.

On the one hand, lactate functions as a signaling molecule in inflammation, with the major pathways, including GPR81, Nuclear Factor Kappa B (NF-κB), and Hypoxia-Inducible Factor 1 Alpha (HIF-1α), interacting to regulate inflammatory processes. In cases of pancreatitis and liver injury, Toll-like receptors (TLRs) recognize bacterial lipopolysaccharide to activate the NF-κB and Mitogen-Activated Protein Kinase (MAPK) signaling pathways, promoting the release of pro-inflammatory cytokines. Lactate inhibits TLRs and pyrin domain-containing protein 3 (NLRP3) inflammasome via the GPR81 pathway, thereby reducing the production of the pro-inflammatory factor interleukin-1β and ultimately decreasing inflammation (13). Additionally, lactate inhibits Yes-associated protein and NF-κB activation through the GPR81 pathway, which subsequently diminishes the production of inflammatory factors in macrophages (14). Furthermore, lactate decreases the nuclear translocation of NF-κB and reduces its activity through HIF-1α, alleviating the microglial inflammatory response (15).

On the other hand, lactate influences the inflammatory response by modulating the function of immune cells. Activated macrophages are classified into two types: M1 and M2, based on their activation state and function. M1 macrophages are primarily responsible for phagocytosing pathogens and secreting pro-inflammatory cytokines, while M2 macrophages play a crucial role in anti-inflammation and tissue repair (16). Phosphofructokinase-2/fructose-2,6-bisphosphatase isoform 3 (PFKFB3) regulates lactate production in endothelial cells. The absence of PFKFB3 in these cells leads to decreased lactate levels, which in turn prevents macrophages from polarizing to the M2 type. Conversely, lactate promotes monocarboxylate transporter 1 (MCT1)-dependent M2 polarization of macrophages, enhancing vascular endothelial growth factor (VEGF) secretion and angiogenesis (17). Additionally, lactate binds to mitochondrial antiviral-signaling protein (MAVS), inhibiting retinoic acid-inducible gene I-like receptor (RLR)-mediated type I interferon production in macrophages, thereby providing resistance to viral infections *in vivo* (18). Lactate not only diminishes pro-inflammatory responses in macrophages by inhibiting pro-inflammatory gene expression but also mediates histone H3K27 acetylation, which further suppresses pro-inflammatory gene transcription (19). Conversely, lactate binds to the prolyl hydroxylase domain-containing 2 (PHD2) catalytic structural domain, inhibiting PHD2 activity and transforming adipose tissue macrophages (ATMs) into a pro-inflammatory profile (20). In studies related to T cells, lactate accumulation at the site of chronic inflammation increases SLC5A12 expression in CD4^+^ T cells, leading to the entry of lactate into the cells and inducing effector phenotypic remodeling. This process is followed by an increase in cytokine interleukin-7 (IL-7) production and enhanced fatty acid synthesis (10). In chronic inflammation, lactate reduces glycolysis necessary for T-cell migration by downregulating hexokinase 1 (HK1), thereby hindering their movement. The production of the pro-inflammatory factor IL-17 by T cells leads to a loss of cytolytic activity (21). In natural killer T (NKT) cells, elevated lactate levels adversely impact NKT cell survival, proliferation, and size, while also reducing the expression of IL-4^+^ and IL-17^+^ (22).

Acute inflammation is a brief immune response by the body to infection or injury. During this response, the body increases lactate production through metabolic reprogramming to meet its energy needs (23). Lactate promotes the inflammatory response by inducing NF-κB expression (23). Conversely, it can also mitigate inflammation and injury in cases of severe acute pancreatitis (13). In chronic inflammation, lactate accumulates at the site of inflammation, leading to intensified inflammatory responses in conditions such as rheumatoid arthritis (10). Overall, lactate plays a complex regulatory role in inflammation. It can exert both anti-inflammatory effects and exacerbate the inflammatory response. Currently, we are unable to define the specific role of lactate in inflammation. Future studies on lactate’s modulation of inflammation could focus on the following questions: How does lactate produce opposing inflammatory effects at various times or in different diseases? Do the pro-inflammatory and anti-inflammatory effects of lactate depend on other factors?

### 2.3 Lactate regulates the tumor microenvironment

The “Warburg effect”, discovered in the 20th century, elucidates the relationship between tumor cells and lactate. This phenomenon suggests that tumor cells preferentially utilize glycolysis as their primary mode of energy metabolism, even in the presence of sufficient oxygen. Consequently, tumor cells tend to produce lactate under aerobic conditions (24). Lactate plays a crucial role in tumors, influencing tumor immunity, signaling, and angiogenesis by modulating the tumor microenvironment (TME).

**Figure 1:**
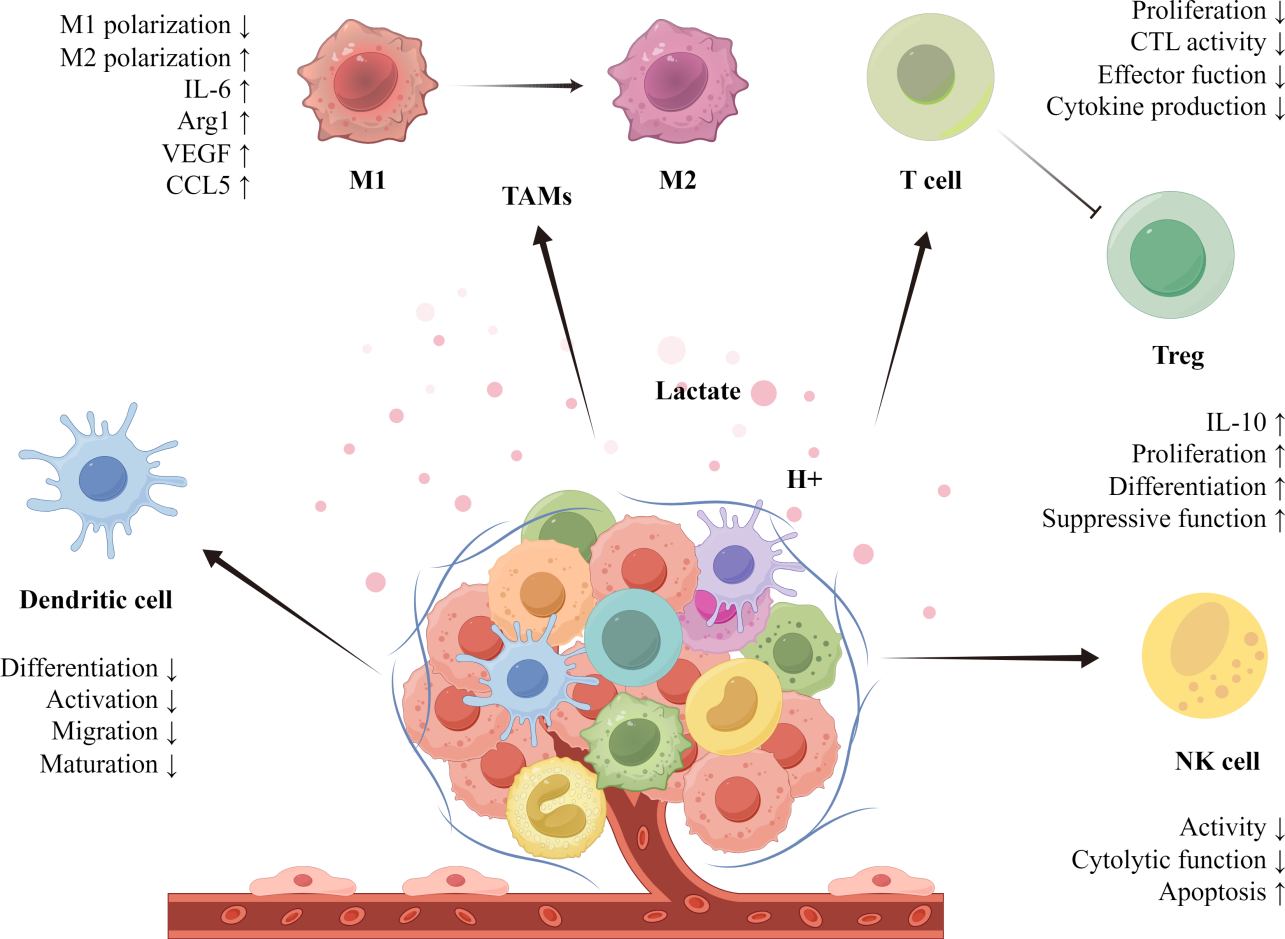
Lactate plays a crucial role in regulating immune cells within the TME. Through metabolic reprogramming, tumor cells release lactate, creating an acidic milieu. This lactate aids tumors in evading immune surveillance and stimulates their growth by modulating the functionality of immune cells. Specifically, lactate drives the polarization of TAMs (tumor-associated macrophages) toward the M2 phenotype, promoting anti-inflammatory actions that enhance tumor progression. Moreover, lactate alters the functions of T cells, Tregs, NK cells, and dendritic cells, enabling immune evasion. The lactate-mediated immunosuppressive network thereby supports tumor growth and infiltration.

Lactate plays a significant immunomodulatory role in the TME, promoting tumor growth by inhibiting immune cell function and facilitating immune evasion (**Figure 1**). In the study of tumor-associated macrophages (TAMs), M1-type macrophages exhibit antitumor effects, while M2-type macrophages promote tumor growth and metastasis. Lactate primarily polarizes TAMs toward the M2 phenotype, contributing to the extensive infiltration of tumor cells in the TME. Lactate derived from HCC cells elevates M2-related markers through the activation of nuclear factor erythroid 2-related factor 2 (Nrf2) in macrophages, thereby promoting M2 phenotypic polarization of TAMs (25). In breast cancer, lactate enhances the M2 phenotype by activating the extracellular signal-regulated kinase (ERK)/signal transducer and activator of transcription 3 (STAT3) signaling pathway, which increases the invasion and growth of breast cancer cells (26). An increase in markers such as interleukin‐6 (IL-6), arginase 1 (Arg1), VEGF, and C-C chemokine ligand 5 (CCL5), along with the stabilization of HIF-1α, further promotes M2 phenotype polarization in TAMs (27). Notably, D-lactate produced by gut microbes can polarize TAMs from the M2 to the M1 phenotype by inhibiting the phosphatidylinositol 3-kinase (PI3K)/protein kinase B (Akt) pathway and activating the NF-κB pathway, thereby inhibiting HCC growth and prolonging the survival of mice (28). Among T cells involved in tumor immunity, lactate inhibits cytotoxic T cell (CTL) function through the p38 MAPK and c-Jun N-terminal kinase (JNK)/c-Jun signaling pathways. Moreover, lactate decreases the production of cytokines and inhibits perforin and granzyme B secretion, resulting in diminished CTL-mediated antitumor immunity (29). Regulatory T cells (Tregs) alter their function and phenotype by increasing programmed death-1 (PD-1) expression through MCT1 uptake of lactate, thereby enhancing their immunosuppressive capacity (30). In natural killer (NK) cells, lactate reduces their cytotoxic activity by inhibiting natural killer cell protein 46 (NKp46) expression (31). Lactate affects NK cell function by interfering with mammalian target of rapamycin (mTOR) signaling and the nuclear translocation of promyelocytic leukemia zinc-finger (PLZF) (32). In studies involving dendritic cells (DCs), plasmacytoid dendritic cells (pDCs) can produce type I interferon to resist tumor immunity. Lactate induces cytoplasmic Ca^2+^ mobilization through GPR81, inhibiting the production of type I IFN and impairing the function of pDCs (33). Additionally, lactate activates the sterol regulatory element-binding protein 2 (SREBP2) pathway in DCs to promote tumor progression (34).

Lactate plays a significant role in tumor angiogenesis through both hypoxia-inducible factor (HIF)-dependent and non-HIF-dependent pathways. In the HIF-dependent pathway, lactate inhibits pyruvate dehydrogenase (PDH) to stabilize HIF‑1α, thereby upregulating angiogenesis‑related genes and promoting new blood‑vessel formation under hypoxic conditions. (35). In the non-HIF-dependent pathway, MCT1 on endothelial cells takes up lactate produced by tumors, subsequently promoting angiogenesis via the activation of the NF-κB/IL-8 signaling pathway (36). Additionally, the N-Myc downstream-regulated gene 3 (NDRG3) protein binds to lactate and c-Raf, facilitating angiogenesis through the RAF-ERK signaling cascade (37).

Lactate serves as a fundamental substance for the metabolic reprogramming of tumor cells. Eukaryotic glycolysis primarily produces L-lactate, while D-lactate is generated by intestinal microorganisms that break down glucose (38). Clinical studies have established a correlation between D-lactate and various diseases. The recent finding that D-lactate inhibits the growth of HCC tumors presents a novel avenue for research. Its potential unique role in disease treatment offers significant opportunities for further investigation. Collectively, the diverse functions of lactate in promoting tumor progression underscore the potential for targeting tumors by disrupting lactate metabolism. Therapeutics aimed at lactate modulation could become a vital component of comprehensive cancer treatment.

## 3 Effects of lactate on liver diseases

### 3.1 Lactate and liver fibrosis

Liver fibrosis is characterized by the abnormal proliferation of connective tissue in the liver, triggered by chronic liver injury (e.g., viral hepatitis, alcoholic liver disease, or NAFLD). When fibrosis progresses to an advanced stage, known as cirrhosis, the liver’s structure is significantly compromised (39). In a healthy liver, hepatic stellate cells (HSCs) remain in a quiescent state. However, upon liver injury, these cells become activated and transform into myofibroblasts, which secrete pro-inflammatory and pro-fibrotic factors, leading to the deposition of extracellular matrix (ECM) (40). The upregulation of glycolytic enzyme PFKFB3 expression is a hallmark of HSC activation, and the RNA-binding protein CPEB4 enhances glycolysis and the level of hepatic fibrosis by upregulating PFKFB3 (41). In liver fibrosis, the Gly28 residue of lactate dehydrogenase A (LDHA) binds to HIF-1α to form a complex, which enhances the expression of glycolysis-related genes, thereby promoting aerobic glycolytic reprogramming during HSC activation. The classic Wnt/β-catenin signaling pathway promotes HSCs glycolysis by upregulating LDHA and HIF-1α, which exacerbates liver fibrosis (42). The lactate receptor GPR81 also plays a significant role in liver fibrosis, lactate enhances the glycolytic activity of HSCs via GPR81, further worsening liver fibrosis. Deletion of GPR81 activates the CREB/Smad7 signaling pathway and reduces liver fibrosis (43). In conclusion, the effects of lactate on liver fibrosis are primarily observed during HSC activation. Increased lactate levels and enhanced glycolysis promote HSCs activation, which exacerbates liver fibrosis. In addition, Kupffer cells, the resident macrophages of the liver, play dual roles in the progression and regression of liver fibrosis (44). HSCs can interact with Kupffer cells, influencing the development of liver fibrosis (45). Lactate may exert either pro-inflammatory or anti-inflammatory effects by affecting the phenotypic transformation of Kupffer cells in the liver, and further experiments are needed to elucidate the exact mechanism involved.

### 3.2 Lactate and NAFLD

NAFLD is a clinicopathological syndrome characterized by excessive intrahepatic triglyceride (TG) deposition due to factors other than alcohol and other well-defined liver injuries (46). Its pathological features are closely associated with obesity, insulin resistance, type 2 diabetes mellitus, and metabolic syndrome (47). In NAFLD, metabolic disorders occur in hepatocytes, including enhanced glycolysis and tricarboxylic acid cycle activity, increased lactate levels, and decreased mitochondrial respiration (48). Research has demonstrated that GPR81 expression is significantly upregulated in fasting mice and downregulated in obese mice. GPR81 knockout mice exhibited increased peripheral adipose tissue decomposition, elevated liver TG levels, and heightened lipid accumulation, resulting in exacerbated liver steatosis. The activation of hepatic GPR81 may represent a potential strategy for the treatment of NAFLD (49). Studies have shown that MCT1 haploinsufficient mice are resistant to liver steatosis. Reduced lactate levels in the liver inhibit sterol regulatory element-binding protein 1 (SREBP1) by activating AMP-activated protein kinase (AMPK), thereby decreasing the lipid accumulation in MCT1 haploinsufficient mice (50). Under high-fat diet conditions, MCT1 knockout mice displayed resistance to obesity, reduced insulin resistance, and no hepatic steatosis (51). In conclusion, lactate is primarily associated with NAFLD through GPR81 and MCT1. Targeting GPR81 and MCT1 in hepatocytes may help reduce liver TG accumulation and alleviate the progression of NAFLD.

### 3.3 Lactate and HCC

HCC is the most common type of primary liver cancer, primarily associated with hepatitis virus infections, cirrhosis, and metabolic liver diseases (52). Abnormal lactate metabolism is a hallmark of HCC, and proton nuclear magnetic resonance (¹H-NMR) spectral analysis has shown elevated levels of lactate in patients with this condition (53).

MCTs are a class of membrane proteins responsible for the transport of lactate and other monocarboxylic acids. Among these, MCT1 and MCT4 have been particularly studied in HCC. MCT1 facilitates the transport of intracellular lactate to the extracellular compartment, while MCT4 performs the opposite function (54). The overexpression of MCT1 and MCT4 in HCC is closely associated with tumor growth, invasion, and metastasis. Autophagy upregulates MCT1 expression through the Wnt/β-catenin signaling pathway, promoting lactate production and HCC metastasis (55). Additionally, the transmembrane protein CD147 inhibits the p53-dependent signaling pathway via MCT1, promoting the reprogramming of glucose metabolism and cell proliferation in HCC cells (56). MCT1-mediated lactate uptake inhibits AMPK, leading to the upregulation of SREBP1 and stearoyl-CoA desaturase-1 (SCD1), which regulates ferroptosis in HCC (57). Furthermore, the upregulation of MCT1 in regulatory T cells increases resistance to anti-PD-1 therapy and correlates with poor prognosis in HCC patients (58). In studies related to MCT4, the inhibition of MCT4 enhances CD8^+^T cell function, thereby suppressing the growth of HCC (59). When MCT4 was knocked down in the HCC cell line (HCCLM3), the expression of Trafficking Protein Particle Complex Subunit 5 (TRAPPC5) was significantly reduced, leading to decreased proliferation and migration (60).

LDHA primarily catalyzes the conversion of pyruvate to lactate and plays a significant role in promoting the progression of HCC by regulating tumor cell metabolism. Various substances have been identified that accelerate HCC progression through the upregulation of LDHA. Notably, high expression levels of methyltransferase NOP2, methyltransferase 5, N6-adenosine (METTL5), and Acylphosphatase 1 (ACYP1) contribute to the growth of HCC by enhancing LDHA expression (61–63). Additionally, protein arginine methyltransferase 3 (PRMT3) facilitates HCC growth by inducing methylation modifications at arginine R112 in LDHA (64). Furthermore, the correlation between LDHA expression levels and HCC prognosis can serve as a predictor of clinical responses to chemotherapy (65).

Lactate transporters (MCT1 and MCT4) and lactate metabolic enzymes (such as LDHA and PKM2) play crucial roles in the progression of HCC. Targeting lactate-associated transporter proteins and enzymes, including MCT inhibitors and LDHA inhibitors, presents a promising therapeutic strategy for the treatment of HCC.

### 3.4 Lactate and liver disease progression

**Figure 2:**
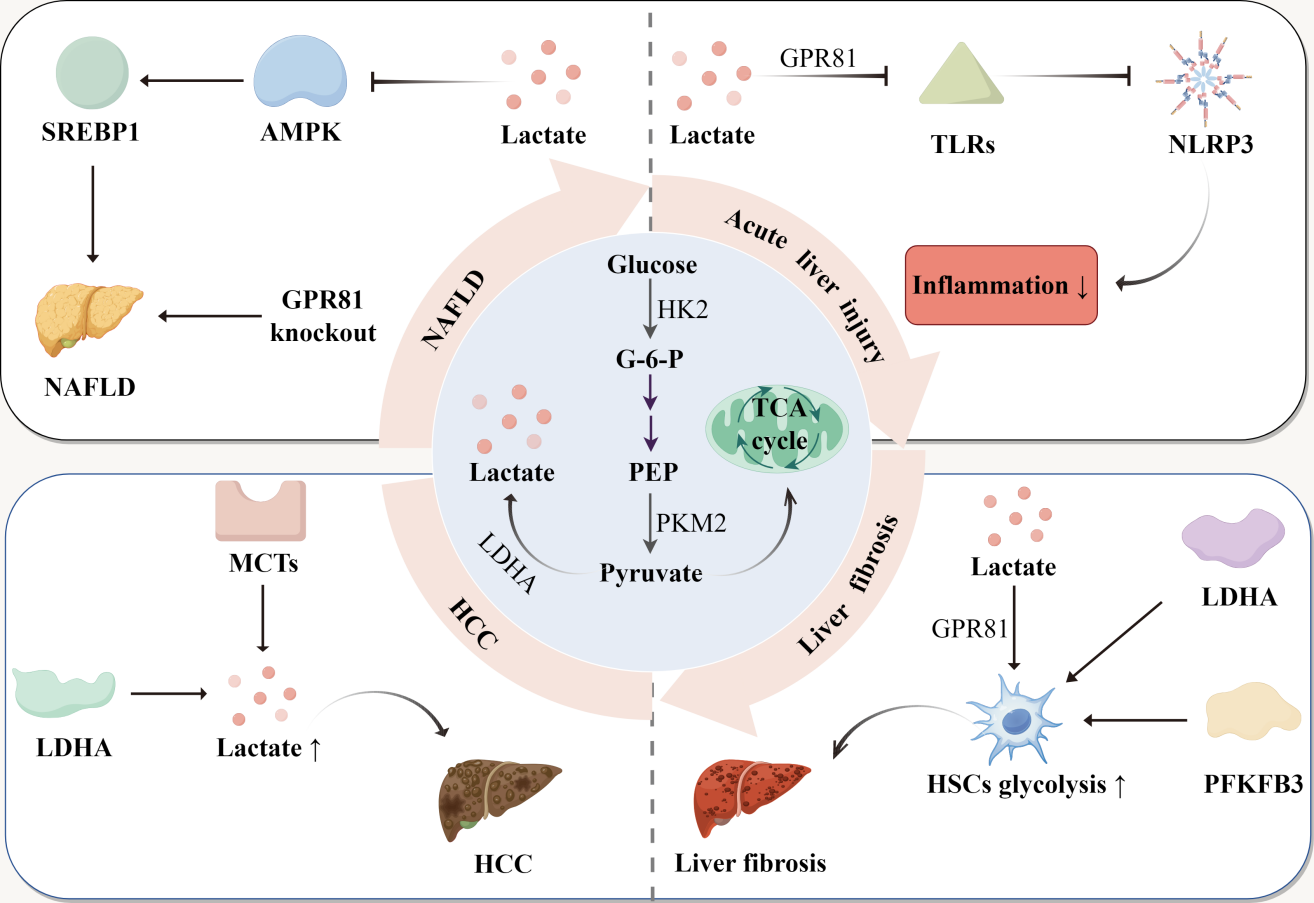
The production of lactate and its role in liver diseases. Glucose is converted to pyruvate, with glucose-6-phosphate (G-6-P) and phosphoenolpyruvate (PEP) as intermediate products. Pyruvate is reduced to lactate or enters the tricarboxylic acid (TCA) cycle. In NAFLD, lactate enhances SREBP1 activity and promotes lipid accumulation by inhibiting AMPK. In addition, GPR81 deletion exacerbates hepatic lipid accumulation. In acute liver injury, lactate inhibits TLRs through GPR81, thereby inhibiting NLRP3 activation and reducing the inflammatory response. In liver fibrosis, lactate and glycolytic enzymes increase glycolysis levels in HSCs, worsening liver fibrosis. In HCC, upregulation of MCTs and LDHA accelerates HCC progression by elevating lactate levels.

The development of liver disease is a progressive process that typically begins with metabolic disorders, gradually deteriorating into chronic conditions, and ultimately leading to liver cirrhosis and HCC (66). NAFLD is primarily caused by metabolic syndrome or a high-fat diet, resulting in hepatocyte steatosis and insulin resistance, which are accompanied by abnormal glucose and fat metabolism (67). Due to these metabolic abnormalities, glycolysis and lactate levels increase (48). Lactate not only regulates lipid deposition and glycolysis by influencing liver metabolism, but the lactate receptor GPR81 and the lactate transporter MCT1 also play significant roles in NAFLD. If fatty liver does not improve over an extended period, it can progress to inflammatory liver diseases, specifically non-alcoholic steatohepatitis (NASH) (68). At this stage, lactate contributes to liver diseases primarily by modulating inflammatory immunity. For instance, lactate mitigates the inflammatory response during acute liver injury through the arrestin β-2/GPR81 pathway, and it reduces inflammation and organ damage in mice with immune-mediated hepatitis (13). If inflammation persists, the condition can advance to liver fibrosis and cirrhosis. Increased glycolysis and the activation of GPR81 further promote the activation of HSCs, thereby exacerbating liver fibrosis. In patients with cirrhosis, some may develop HCC (69). Liver cancer cells enhance lactate production through the “Warburg effect”, and lactate helps them evade the immune system, and promotes angiogenesis by modulating the TME. Consequently, lactate plays a crucial role throughout the entire spectrum of liver disease, and lactate-targeted therapeutic strategies may offer a new breakthrough in the treatment of liver diseases (**Figure 2**).

## 4 Regulatory enzymes involved in lactylation

Lactylation modification is classified into two categories: enzyme-dependent and non-enzyme-dependent (70). Enzyme-dependent lactylation relies on specific catalytic enzymes, whereas non-enzyme-dependent lactylation primarily depends on D-lactate (70). Research has predominantly focused on enzyme-dependent lactylation, with comparatively less attention given to non-enzyme-dependent lactylation. The regulatory enzymes involved in lactylation are mainly categorized into three groups: lactyltransferase (“Writer”), delactylase (“Eraser”) and lactylation-recognizing protein (“Reader”), all of which play crucial roles in the lactylation process (71) (**Table 1**).

**Table 1:** Enzymes of lactylation.

Function	Enzymes	Reaction substrate	Species	Subcellular location	(Refs.)
Writer	p300	Lactyl-CoA	Homo sapiens	Cell nucleus	(6)
	CBP	Lactyl-CoA	Homo sapiens	Cell nucleus	(73)
	AARS1	Lactate	Homo sapiens	Cytoplasm	(75, 76)
	AARS2	Lactate	Homo sapiens	Mitochondrion matrix	(75, 77)
	KAT8	Lactyl-CoA	Homo sapiens	Cell nucleus	(143)
	GCN5	Lactyl-CoA	Homo sapiens	Cell nucleus	(89)
	KAT5	Lactyl-CoA	Homo sapiens	Cell nucleus	(114)
	KAT7	Lactyl-CoA	Homo sapiens	Cell nucleus	(144)
Eraser	HDAC1	Histones	Homo sapiens	Cell nucleus	(78)
	HDAC2	Histones	Homo sapiens	Cell nucleus and cytoplasm	(78)
	HDAC3	Histones	Homo sapiens	Cell nucleus and cytoplasm	(78, 80)
	SIRT1	Histones	Homo sapiens	Cell nucleus and cytoplasm	(81)
	SIRT2	Histones	Homo sapiens	Cell nucleus and cytoplasm	(145)
	SIRT3	Histones	Homo sapiens	Mitochondrion matrix	(81)
Reader	Brg1	ATP	Homo sapiens	Cell nucleus	(146)

Writer is primarily responsible for transferring lactate groups to amino acid residues on target proteins. Notable examples of these enzymes include p300, CBP (CREB-binding protein), KAT8, alanyl-tRNA synthetase 1/2 (AARS1/2), and GCN5. p300 is not only a classical histone acetyltransferase but also a significant lactylation-writer (72). Zhang et al. discovered that lactylation levels in HEK293T cells increased upon the overexpression of p300. Conversely, knocking down p300 resulted in decreased lactylation levels, thereby confirming its role in lactylation writing (6). During sepsis, macrophages enhance the lactylation of the high mobility group box-1 (HMGB1) via p300/CBP, which damages endothelial cells and promotes the progression of sepsis (73). Additionally, lactate elevates H3K18 lactylation levels through p300/CBP (74). In contrast to p300, AARS1/2 catalyzes lactylation without the involvement of Lactyl-CoA and can directly utilize lactate for lactylation modifications (75). AARS1/2 senses lactate levels to regulate lactylation modifications and uses ATP to mediate the lactylation of cyclic GMP-AMP synthase (cGAS) (75). The p53 protein is a tumor-suppressive factor that plays a crucial role in DNA repair and cell cycle arrest. AARS1 catalyzes the lactylation of the K120 and K139 sites in the p53 DNA-binding structural domain, contributing to tumorigenesis (76). Under hypoxic conditions, AARS2 catalyzes the lactylation of PDHA1 and carnitine palmitoyltransferase 2 (CPT2), regulating the production of Ac-CoA (Acetyl-Coenzyme A) and oxidative phosphorylation (OXPHOS) (77).

“Eraser” removes lactylation modifications by eliminating the lactate moiety, thereby restoring the protein to its original state. Moreno-Yruela et al. first discovered that class I Histone Deacetylases (HDAC1-3) and Sirtuin1-3 (SIRT1-3) exhibit delactylation modification activity and proposed HDAC3 as the most potent delactonase *in vitro* (78). The overexpression of HDAC in endothelial cells reduces the level of H3K9 lactylation and inhibits neoangiogenesis (79). In atherosclerosis, HDAC3 overexpression decreases the level of H4K12 lactylation and the expression of senescence-associated secretory phenotype (SASP), preventing vascular smooth muscle cell senescence (80). Overexpression of SIRT1 and SIRT3 from the sirtuin family reduces the overall level of lactylation and modulates ENO1-K228 lactylation. Experiments have confirmed that SIRT1 de-lactylates pyruvate kinase M2 (PKM2) at K207, while SIRT3 has a lesser effect on it (81). This modification further suggests that SIRT1 and SIRT3 act on different target sites (81).

“Reader” is a protein that can recognize lactylation, and specifically bind to the lactylation group domain (82). Currently, research related to “Reader” remains limited (83). In conclusion, the regulatory enzymes involved in lactylation play significant roles in the lactylation of enzymatic reactions, and further experimental exploration of the mechanisms regulating lactylation-modifying enzymes is warranted.

## 5 Biological functions of lactylation

### 5.1 Lactylation regulates inflammation and immunity

Lactylation plays an important role in biological processes as an emerging post-translational modification of proteins. In regulating inflammation and immunity, it not only mediates inflammatory responses by affecting the function of immune cells, but also regulates the expression of inflammation-related genes.

In macrophage-related studies, lactylation promotes the polarization of M2-type macrophages, playing a crucial role in anti-inflammation and tissue repair. PKM2 is a key regulator of metabolism and inflammation, and lactate activates PKM2 by promoting its lactylation at the K62 site, facilitating the shift from a pro-inflammatory to a reparative phenotype in macrophages (84). The polarization state of macrophages correlates with mitochondrial morphology, with M1-type macrophages exhibiting a longer mitochondrial network, while M2-type macrophages display a shorter network. Mitochondrial fragmentation alters lactate metabolism, and accumulated lactate promotes the M2 phenotype in macrophages and inflammatory regression by enhancing lactylation modifications and Arg1 expression (85). In chronic colitis, the expression of B-cell adapter for PI3K (BCAP) in macrophages increases histone lactylation of repair genes, promoting the transition of inflammatory macrophages to an M2 phenotype, which contributes to tissue repair and wound healing (86). In addition, microglia are intrinsic immune cells within the central nervous system, and the progression of Alzheimer’s disease is closely associated with microglia-mediated inflammatory responses. In 5XFAD (B6SJL-Tg) mice, there are elevated levels of H4K12 lactylation in plaque-adjacent microglia, which further activates glycolysis-associated genes, creating a positive feedback loop involving glycolysis, H4K12, and PKM2 that can lead to dysfunctional microglia (87).

Lactylation also regulates the expression of inflammation-related genes by affecting histones. In sepsis-associated acute kidney injury (SA-AKI), the overall level of lactylation in renal tubular epithelial cells (RTECs) and the level of H3K18 lactylation (H3K18la) were found to be increased. The BAY-876-induced reduction of H3K18la suppressed the expression of inflammatory factors such as IL-6 and tumor necrosis factor α (TNF-α), leading to improved renal function in SA-AKI (88). During inflammation resolution and cardiac repair following after myocardial infarction (MI), histone lactylation modification not only promotes the activation of repair genes such as Lrg1, Vegf-α, and IL-10, but also facilitates the establishment of a conducive repair environment and cardiac functional remodeling (89).

In conclusion, studies on lactylation modification-mediated inflammation have primarily focused on the polarization of macrophage. In various diseases, lactylation not only exerts anti-inflammatory effects, as seen in chronic colitis, but also exacerbates inflammatory responses (such as SA-AKI). Furthermore, the regulatory effect of lactylation on the immune system is often referred to as the “lactate clock”, indicating the time-dependent regulation of lactylation on inflammatory responses (23). The specific mechanisms by which lactylation influences inflammation require further investigation, with the expectation of providing new strategies for treating inflammatory diseases.

### 5.2 Lactylation regulates protein function

Lactylation regulates both histones and non-histone proteins (4). Histone lactylation influences gene expression through epigenetic mechanisms, while non-histone lactylation modulates protein function through steric hindrance, conformational changes, and charge neutralization (90). In this discussion, we primarily focus on how non-histone lactylation alters protein function by affecting protein-molecule interactions, protein stability, and nuclear translocation.

Lactylation regulates protein-molecule interactions, including protein-protein, protein-DNA, and protein-RNA interactions. Lactylation of α-Myosin heavy chain (α-MHC) at K1897 promotes its binding to Titin. In contrast, α-MHC K1897R mutant mice exhibit reduced lactylation and decreased binding to Titin, which exacerbates heart failure in these mice (91). Lactylation of heterochromatin protein CBX3 at K10 enhances its binding to H3K9me3 in cancer cells, contributing to the progression of gastrointestinal cancer (92). Compared to the MRE11 (homologous recombination protein) K673R mutant, wild-type MRE11 demonstrates a higher affinity for DNA, suggesting that MRE11 lactylation enhances its DNA-binding capability (93). In intrahepatic cholangiocarcinoma, lactylation of nucleolin at K477 alters the structure of the nucleolin and regulates its binding to the mRNA of the MAP kinase-activated death domain protein (MADD), thereby preventing premature termination of translation (94).

Lactylation modulates protein stability by inhibiting ubiquitin-mediated degradation, altering protein spatial conformation, and neutralizing charge. Transcription Factor EB (TFEB) plays a crucial role in autophagy and lysosomal gene expression, and lactate promotes TFEB expression alongside increased levels of TFEB lactylation. The E3 ubiquitin ligase WWP2 mediates the ubiquitination and proteasomal degradation of TFEB. Lactylation of TFEB at the K91 site prevents the interaction between TFEB and WWP2, thereby enhancing TFEB stabilization and inhibiting its degradation (95). Overexpression of Aldolase B (ALDOB) increases lactate and lactylation levels, which in turn activates the cell adhesion molecule 6 (CEACAM6) and enhances its stability (96). In cervical cancer, L-lactate not only increases the expression of Discoidin, CUB, and LCCL domain-containing type I (DCBLD1) via HIF-1α but also directly mediates DCBLD1 lactylation. DCBLD1 lactylation at the K172 site regulates its stability and inhibits its ubiquitination (97).

Protein nuclear translocation is the process of transferring proteins from the cytoplasm to the nucleus, and lactylation can influence this translocation to regulate protein function. In rheumatoid arthritis, artemisinin (ART) promotes p300-dependent lactylation of PKM2 and inhibits its nuclear translocation (98). In pancreatic cancer, L-lactate-mediated lactylation of NMNAT1 at K128 enhances its nuclear translocation and activity, thereby maintaining NAD^+^ homeostasis in the nucleus (99). Heat shock protein A12A (HSPA12A) promotes glycolysis and lactate production by increasing the levels of the transcription factor HIF-1α. Additionally, lactate elevates the level of c-Myc lactylation in renal tubular epithelial cells, which further induces an increase in the nuclear localization of the transcriptional factor c-Myc (100).

### 5.3 Lactylation regulates metabolism

Lactylation is an emerging marker of metabolic reprogramming (101). Myocardial ischemia-reperfusion injury (MIRI) is closely linked to metabolic reprogramming, and dexmedetomidine (Dex) has been shown to alleviate MIRI by modulating glycolysis. This process reduces lactate levels and Malate Dehydrogenase 2 (MDH2) K241 lactylation, thereby enhancing mitochondrial function and mitigating ferroptosis (102). Lactate accumulation poses a significant risk following skin flap transplantation, as PKM overexpression promotes abnormal glycolysis and elevates lactate levels. This accumulated lactate induces Twist1 lactylation and its nuclear translocation, driving the progression of endothelial-mesenchymal transition (EndoMT) via increased transforming growth factor-β (TGF-β) expression (103). Kynurenine metabolism, the primary catabolic pathway of tryptophan, plays a crucial role in cardiovascular diseases. X-ray-induced lactylation of protein disulfide-isomerase (P4HB) at the K311 site, in conjunction with aloe rhodopsin, inhibits the lactylation of P4HB and mitochondrial autophagy. This stabilizes aspartate aminotransferase glutamate-oxaloacetate transaminase 2 (GOT2)-mediated kynurenine metabolism and alleviates radiation-induced cardiac injury (104). In conclusion, studies on the regulation of metabolism by lactylation have predominantly focused on glycolysis, whereas research into the regulation of amino acid metabolism, lipid metabolism, and oxidative phosphorylation remains relatively limited.

### 5.4 Lactylation regulates the tumor microenvironment

The role of lactate-mediated lactylation in tumors has been extensively investigated in recent years. Lactylation not only aids in immune escape within tumors but also plays a crucial role in regulating tumor cell proliferation and migration, metabolic reprogramming, and influencing tumor therapy.

In colon cancer cells, the knockdown of proprotein convertase subtilisin/kexin type 9 (PCSK9) reduces both macrophage migration inhibitory factor (MIF) expression and lactylation levels, thereby promoting macrophage M1-type polarization (105). Lactate enhances Treg function through MOESIN lactylation and activates the TGF-β signaling pathway, thereby promoting tumor growth (106). In tumor-infiltrating myeloid cells (TIMs), lactate accumulated in TME not only increases the expression of methyltransferase-like 3 (METTL3) in TIMs through lactonization, but also induces immunosuppression in TIMs (107). Beyond its role in regulating immune cell function and influencing tumor immunity, it has been reported that L-lactate diminishes natural immune responses by lactylating the nucleotidyltransferase cGAS, thereby reducing its activity (75).

In studies focused on tumor cell proliferation and migration regulation, the lactate transferase AARS1 directly binds to lactate, catalyzing the lactylation of the oncogene p53. This process attenuates the oncogenic effects of p53 and promotes tumor proliferation (76). In renal clear cell carcinoma, inactivated VHL-mediated histone lactylation activates platelet-derived growth factor receptor β (PDGFRβ) expression, thus enhancing cancer cell proliferation and migration (108). In HCC, lactate-induced lactylation of histones H3K9 and H3K56 in HCC cells boosts their proliferative capacity (109). Furthermore, lactylation at the K28 site of adenylate kinase 2 (AK2) facilitates the proliferation and invasion of HCC cells (110).

Tumor metabolic reprogramming involves the adaptation of tumor cells to meet the demands of tumor growth by altering their metabolism. Tumor cells enhance glycolysis to promote lactate accumulation and its associated modifications, playing a crucial role in metabolic reprogramming. For instance, lactylation at the K76 site of Insulin-like growth factor 2 mRNA-binding protein 3 (IGF2BP3) enhances the expression of phosphoenolpyruvate carboxykinase (PCK2) and promotes serine metabolic reprogramming in HCC (111). In pancreatic ductal adenocarcinoma, Nucleolar and spindle associated protein 1 (NUSAP1) lactylation at H3K18 activates mitotic spindle assembly checkpoint regulator TTK and BUB1B, which further promotes glycolysis and lactate accumulation in tumor cells (112). In colorectal cancer, tumor-derived lactate induces glucose metabolism reprogramming and cancer progression by establishing an NSUN2/YBX1/m^5^C-ENO1 positive feedback loop through the lactylation of the m^5^C methyltransferase NSUN2 (113). Analysis of the lactateome reveals that lactylated proteins are significantly enriched in carbohydrate, fatty acid, and amino acid metabolism in HCC, suggesting that lactylation may influence HCC progression by regulating various metabolic processes (110).

It is also noteworthy that lactylation can impact the chemotherapeutic efficacy and drug resistance of tumors. Lactylation of the NBS1 protein at the K388 site promotes the formation of MRN complexes, which facilitates DNA repair and reduces damage during radiotherapy (114). In glioblastoma, lactylation of DNA damage repair-related protein (XRCC1) at the K247 site enhances its binding affinity to the nuclear transporter protein importin α and promotes DNA damage repair in cancer cells (115). Acetyltransferase CBP-mediated lactylation of MRE11 not only facilitates DNA excision and homologous recombination repair, but also increases chemotherapy resistance (93). In non-small cell lung cancer (NSCLC), lactylation of apolipoprotein C-II (APOC2) at the K70 site elevates extracellular lipolysis and levels of free fatty acids, contributing to tumor cell metastasis and immunotherapy resistance (116). Additionally, lactylation of IGF2BP3 enhances S-adenosylmethionine (SAM) synthesis and RNA m6A modification, fostering therapeutic resistance, particularly lenvatinib resistance in HCC (111).

## 6 Effects of lactylation on liver diseases

### 6.1 Lactylation and liver fibrosis

In liver fibrosis, lactylation mediates the progression of hepatic fibrosis primarily by influencing the activation of HSCs. The enzyme Hexokinase 2 (HK2) induces elevated lactate levels and H3K18 lactylation, thereby participating in the activation process of HSCs, which includes affecting their gene expression and metabolic regulation. Deletion of the HK2 gene inhibits HSC activation, suggesting that targeting HK2 and H3K18 lactylation may represent a potential therapeutic approach for hepatic fibrosis (117). The m^6^A-binding protein IGF2BP2 exhibits increased expression in liver fibrosis and activated HSCs and correlates with poor prognosis. IGF2BP2 promotes liver fibrosis progression by increasing aldolase A (ALDOA) expression and overall lactylation levels (118). SOX9, is a transcription factor that plays a specific role in liver fibrosis. It was found that knockdown of LDHA reduced H3K18 lactylation levels by decreasing SOX9 transcription, which in turn diminished HSC activation and extracellular matrix deposition, and alleviated liver fibrosis in rats (119). In conclusion, inhibiting HSC activation by targeting lactylation represents a promising strategy for the treatment of liver fibrosis in the future.

### 6.2 Lactylation and NAFLD

NAFLD is a complex metabolic disorder characterized by abnormal hepatic lipid metabolism and excessive intracellular lipid accumulation in hepatocytes (120). Lactylation plays a significant role in the progression of NAFLD. Research has demonstrated that lactylation regulates lipid accumulation in NAFLD. In studies involving MPC1 knockout mice, increased levels of lactate and lactylation in the liver were observed due to the specific mechanism by which the MPC1 gene mediates the lactylation of the K673 site on fatty acid synthase (FASN). This process results in reduced FASN activity and lipid accumulation. Therefore, drugs targeting the MPC1 gene related to FASN lactylation may offer a promising therapeutic strategy for NAFLD (121). Additionally, lactylation can also influence NAFLD by modulating inflammatory responses and energy metabolism. However, the exact mechanisms require further investigation.

### 6.3 Lactylation and HCC

Recent studies have demonstrated that lactate-mediated lactylation is closely linked to HCC (122). These modifications promote HCC cell proliferation and progression by regulating metabolic pathways, the expression of key proteins, and related enzymes. Liver cancer stem cells (LCSCs) possess the ability to differentiate into various HCC cell types, contributing to HCC heterogeneity. It has been found that lactylation of the H3K56 site in LCSCs not only regulates cell stemness but also correlates with cell proliferative capacity. Additionally, lactylation at K230/322 in ALDOA enhances LCSCs stemness via dead box deconjugate enzyme 17 (DDX17) (123). Acetyltransferase p300 catalyzes acetylation of pyruvate dehydrogenase complex component X (PDHX) at the Lys 488 site, which in turn promotes lactylation of H3K56 and drives oncogene expression, mediating HCC progression (124). The expression of Pyrroline-5-carboxylate reductase 1 (PYCR1) is increased in HCC, and knockdown of PYCR1 reduces IRS1 histone activity by down-regulating H3K18 lactylation, thereby inhibiting HCC proliferation (125). Non-histone ABCF1 lactylation at the K430 site promotes HCC cell proliferation and migration through the HIF-1α signaling pathway. The study also identified key enzymes for lactylation, including p300 as a lactyltransferase and HDAC1/HDAC3 as delactylases (126). The expression of CENPA, a variant of histone H3, was increased in HCC, and lactylation of CENPA at the K124 site enhanced its transcriptional activity and promoted HCC progression (127). Lactylation promotes the occurrence of HCC by regulating various biological processes. Lactylation levels serve as a biomarker for HCC, useful for early detection and prognosis assessment (122). Future research and targeted intervention on lactylation may provide novel strategies for the diagnosis and treatment of HCC.

### 6.4 Lactylation and liver disease progression

**Figure 3:**
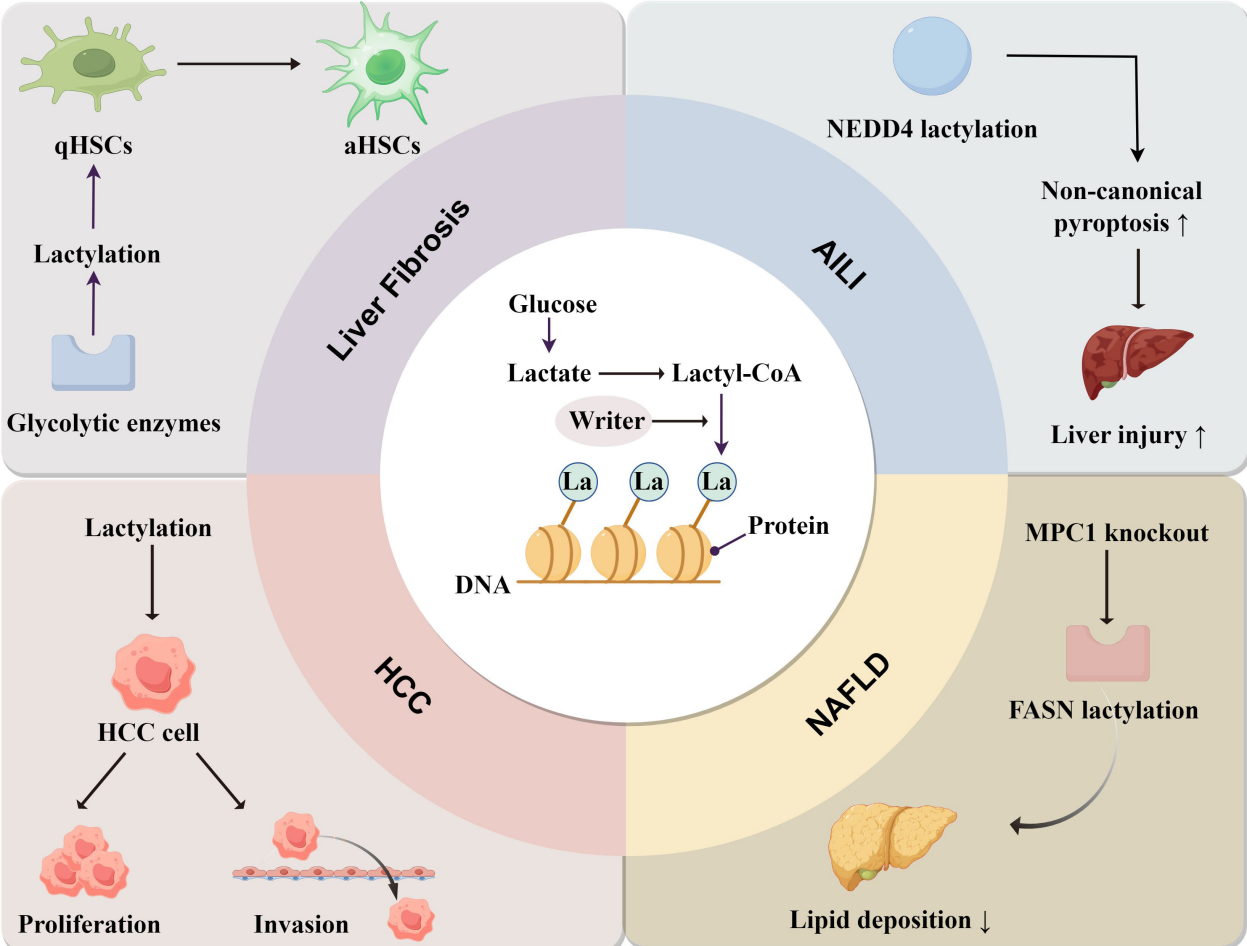
The mechanism of lactylation and its role in liver diseases. Lactate derived from glucose is converted into Lactyl-CoA, and the lactate group is added to the proteins under the action of “Writer”. In liver fibrosis, lactylation is involved in the transformation of quiescent hepatic stellate cells (qHSCs) into activated hepatic stellate cells (aHSCs). In HCC, lactylation enhances the proliferation and invasion of cancer cells. In NAFLD, lactylation mainly reduces lipid accumulation. In AILI, NEDD4 lactylation promotes non-canonical pyroptosis pathways and exacerbates liver injury.

Lactylation is a key regulator in the development of liver diseases, but the specific molecular mechanism and principle of action still need further verification and in-depth discussions. In NAFLD, lactylation of fatty acid synthase inhibits its own activity and reduces lipid accumulation in the liver, suggesting that lactylation appears to improve NAFLD, but further research is needed. In APAP-induced liver injury (AILI), NEDD4 (a negative regulator of Caspase-11) lactylation reduces the ubiquitination capacity of Caspase-11 and upregulates non-canonical pyroptosis pathways, exacerbating hepatic injury in the context of AILI (128). In liver fibrosis, glycolytic enzymes can promote histone lactylation, thus promoting HSC activation and liver fibrosis. Therefore, targeting glycolytic enzymes and lactylation represents a potential therapeutic strategy for liver fibrosis. Lactylation of many key proteins plays an important role in the progression of HCC when liver diseases advance to irreversible stages. Moreover, lactylation is a novel anti-cellular cancer strategy. In summary, lactylation plays a complex role in the progression of liver diseases, as it can both promote disease development by regulating metabolism and immunity, and in some cases, exert a protective role (**Figure 3**).

## 7 Lactate and lactylation in the treatment of liver diseases

### 7.1 Regulating lactate metabolism for the treatment of liver diseases

Based on the various roles of lactate in liver diseases, it demonstrates significant potential for the treatment of these conditions. In liver fibrosis, curcumol disrupts the KLF5/LDH-A positive feedback loop, while reducing extracellular matrix deposition and inhibiting angiogenesis and the progression of liver fibrosis in liver sinusoidal endothelial cells (LSECs) (129). Quercetin modulates the glycolysis of LSECs and decreases neutrophil infiltration, thereby alleviating hepatic injury and fibrosis by downregulating the expression of HK2, phosphofructokinase platelet (PFKP), and PKM2, as well as lactate production (130). Costunolide inhibits glycolysis-related HSCs activation by inhibiting HK2 expression, thereby reducing liver fibrosis (131). Rotundic acid (RA), a triterpenoid primarily found in the bark of iron holly, is commonly used to treat cardiovascular disease and hepatitis. A study indicated that RA reduces inflammation and adipogenesis in NASH by inhibiting glycolysis and lactate production, and it also mitigates inflammation by modulating the TLR4/AP1 signaling pathway (132). In NAFLD, Metformin has been shown to improve hepatic steatosis, fatty infiltration, and hepatic triglyceride levels in a GPR81-dependent manner (49). In acute liver failure, Galloflavin reduced liver damage and apoptosis via inhibition of LDH and pyruvate dehydrogenase complex (PDHC), and decreased hepatic cytokine expression and improved survival (133). Lenvatinib is a therapy for HCC. Tumor-derived lactate induces programmed cell death-1 ligand (PD-L1^+^) neutrophils and PD-L1 expression, thereby inhibiting the efficacy of lenvatinib. Celecoxib enhances the effects of lenvatinib by reducing PD-L1^+^ neutrophil infiltration and PD-L1 expression (134). OR protein hydrolysate (ORPH) inhibits the post-transcriptional expression of PKM2 through the upregulation of miR-491-5p, thereby suppressing glycolysis and exerting anti-tumor effects in Hep3B cells (135). In summary, drug development targeting lactate mainly focuses on two approaches: preventing lactate production by using glycolytic enzyme inhibitors, or inhibiting lactate transport by modulating lactate transporters (**Table 2**).

**Table 2:** Drugs that regulate lactate metabolism in liver diseases.

Disease	Drug	Mechanism	(Refs.)
Liver fibrosis	Curcumol	Interrupts the KLF5/LDH-A positive feedback loop	(129)
	Quercetin	Decreases the expression of glycolysis-related enzymes	(130)
	Costunolide	Reduces HSCs activation via inhibition of HK2	(131)
NAFLD	Metformin	Improvement of lipid deposition	(49)
NASH	Rotundic acid	Inhibits glycolysis and lactate production	(132)
Acute liver failure	Galloflavin	Reduces liver damage via inhibition of LDH and PDHC	(133)
HCC	Celecoxib	Decreases PD-L1^+^ neutrophil infiltration and PD-1 expression	(134)
	ORPH	Regulates miR-491-5p/PKM2 axis	(135)

### 7.2 Regulating lactylation for the treatment of liver diseases

In recent years, research into lactylation in liver diseases has made significant strides, providing fresh insights into potential therapeutic approaches. Huazhuo Tiaozhi granule (HTG), a traditional Chinese herbal medicine, promotes histone lactylation in hepatocytes, effectively alleviating dyslipidemia (136). During hepatic injury, histone lactylation levels are elevated in Kupffer cells. Salvianolic acid B attenuates histone lactylation in Kupffer cells, thereby suppressing their M1 polarization and consequently mitigating liver injury and fibrosis in murine models (137). Tectorigenin (TEC) facilitates the interaction between TEC-mediated tRF-31R9J and HDAC1, inhibiting histone lactylation and acetylation in ferroptosis-related genes, which reduces ferroptosis in hepatocytes and lipid accumulation in NASH (138). High expression of HSPA12A suppresses HMGB1 lactylation in hepatocytes, reducing HMGB1 secretion and macrophage activity, thereby decreasing Liver ischemia/reperfusion (LI/R) (139). Several natural compounds combat HCC by inhibiting histone lactylation. Royal jelly acid and the triterpene antitumor compound demethylzeylasteral have been shown to suppress HCC cell proliferation by inhibiting H3 lactylation (140, 141). Honokiol prevents HCC growth by de-lactylating the cyclin E2 through the activation of SIRT3 (142). The small molecule drug tubuloside A (TubA) inhibits the progression of HCC by affecting the lactylation of non-histone ABCF1 (126). Additionally, the enzymes and proteins associated with lactylation may serve as potential targets for the treatment of liver diseases. In the future, targeting lactylation-related proteins or enzymes to reduce lactylation levels could emerge as a novel therapeutic strategy for liver diseases (**Table 3**).

**Table 3:** Drugs that regulate lactylation in liver diseases.

Disease	Drug	Mechanism	(Refs.)
Liver fibrosis	Salvianolic acid B	Inhibits the histone lactylation of macrophages by downregulating the level of LDHA	(137)
NAFLD	HTG	Increases histone lactylation to improve blood lipids	(136)
NASH	Tectorigenin	Inhibits histone lactylation and acetylation in ferroptosis-related genes	(138)
LI/R	HSPA12A	Inhibits HMGB1 lactylation and secretion	(139)
HCC	Royal jelly acid	Inhibits H3 histone lactylation	(140)
	Demethylzeylasteral	Inhibits H3 histone lactylation	(141)
	Honokiol	Activates SIRT3 to make cyclin E2 delactylation	(142)
	TubA	Inhibits lactylation in ABCF1	(126)

## 8 Conclusions and perspectives

In this review, we systematically describe the biological functions of lactate and lactylation, and summarize their roles in liver fibrosis, NAFLD and HCC. Lactate exerts its biological effects primarily through interactions with specific receptors and enzymes, such as GPR81, MCTs, and LDH, among which GPR81 is particularly important. Lactylation refers to the enzymatic addition of lactate group to specific modification sites on proteins, thereby influencing biological functions. Unlike earlier reviews that focused exclusively on lactate or lactylation, we highlight the emerging roles of both lactate and lactylation in liver diseases. While lactylation has predominantly been studied in the context of histones, we propose specific mechanisms for lactylation of non-histones. Furthermore, the impact of lactylation on tumor chemotherapy and drug resistance warrants attention.

Lactate and lactylation are closely linked to liver diseases, but the specific molecular mechanisms and biological functions require further exploration. The identification of therapeutic targets and drugs could provide novel insights for innovative liver disease treatments. Lactylation research holds immense potential, including the discovery of additional lactylation-related enzymes, elucidation of its dual role in inflammation, and clarification of its mechanisms within the TME. Future clinical studies on lactylation will likely focus on assessing whether lactylation levels can serve as a disease biomarker, and on developing lactylation-targeted drugs for cancer and immunotherapy.
